# Impact of Ga^3+^ Ions on the Structure, Magnetic, and Optical Features of Co-Ni Nanostructured Spinel Ferrite Microspheres

**DOI:** 10.3390/nano12162872

**Published:** 2022-08-21

**Authors:** Munirah A. Almessiere, Yassine Slimani, Sadaqat Ali, Abdulhadi Baykal, Rabindran Jermy Balasamy, Sadik Guner, İsmail A. Auwal, Alex V. Trukhanov, Sergei V. Trukhanov, Ayyar Manikandan

**Affiliations:** 1 Department of Biophysics, Institute for Research and Medical Consultations (IRMC), Imam Abdulrahman Bin Faisal University, P.O. Box 1982, Dammam 31441, Saudi Arabia; 2 Department of Physics, College of Science, Imam Abdulrahman Bin Faisal University, P.O. Box 1982, Dammam 31441, Saudi Arabia; 3 Mechanical and Energy Engineering Department, College of Engineering, Imam Abdulrahman Bin Faisal University, P.O. Box 1982, Dammam 31441, Saudi Arabia; 4 Department of Nanomedicine Research, Institute for Research and Medical Consultations (IRMC), Imam Abdulrahman Bin Faisal University, P.O. Box 1982, Dammam 31441, Saudi Arabia; 5 Institute of Inorganic Chemistry, RWTH Aachen University, 52074 Aachen, Germany; 6 Department of Chemistry, Sule Lamido University, Kafin Hausa 731, Nigeria; 7 Smart Sensors Laboratory, Department of Electronic Materials Technology, National University of Science and Technology (MISiS), 119049 Moscow, Russia; 8 Laboratory of Magnetic Films Physics, SSPA Scientific and Practical Materials Research Centre of NAS of Belarus, 19, P. Brovki Str., 220072 Minsk, Belarus; 9 Department of Chemistry, Bharath Institute of Higher Education and Research, Bharath University, Chennai 600073, Tamil Nadu, India

**Keywords:** nanospinel ferrites, nanocrystalline structure, BET analysis, magnetic features

## Abstract

Co-Ni ferrite is one of the crucial materials for the electronic industry. A partial substitution with a rare-earth metal brings about modification in crystal lattice and broadens knowledge in the discovery of new magnetic material. Current work reports a Ga^3+^ substitution in the Co-Ni ferrite with composition Co_0.5_Ni_0.5_Fe_2−x_Ga_x_O_4_ (where x = 0.0, 0.2, 0.4, 0.6, 0.8, and 1.0), herein referred to as spinel ferrite microspheres (CoNiGa-SFMCs). The samples were crystallized hydrothermally showing a hollow sphere morphology. The crystal phase, magnetic, morphology, and optical behaviour were examined using various microscopy and spectroscopic tools. While the XRD confirmed the phase of SFMCs, the crystallite size varied between 9 and 12 nm. The Tauc plot obtained from DRS (diffuse reflectance spectroscopy) shows the direct optical energy bandgap (*E*_g_) of the products, with the pristine reading having the value of 1.41 eV *E*_g_; the band gap increased almost linearly up to 1.62 eV along with rising the Ga^3+^ amount. The magnetic features, on the other hand, indicated the decrease in coercivity (H_c_) as more Ga^3+^ is introduced. Moreover, there was a gradual increase in both saturation magnetization (M_s_) and magnetic moment ( $n_{B}$ ) with increasing amount of Ga^3+^ till x = 0.6 and then a progressive decline with increases in the x content; this was ascribed to the spin-glass-like behavior at low temperatures. It was detected that magnetic properties correlate well with crystallite/particle size, cation distribution, and anisotropy.

## 1. Introduction

Complex iron oxides with different structures currently attract great attention [1,2,3,4]. Spinel ferrites (MFe_2_O_4_) are complex oxides that crystallize via FCC and have a [(M^2+^)(Fe_2_^3+^)O_4_] spinel structure. This type of ferrite is most intensively investigated among the other ferrites [5,6,7,8,9,10]. Based on the Fe^3+^ (trivalent) and M^2+^ (divalent) ion distribution between the octahedral (O_h_) sites and tetrahedral (T_d_) sites, they are called inverse and normal ferrites with spinel-like structures [11,12]. The spinel ferrite structure has been extensively studied because of its innate properties in electrical and magnetic fields [13,14]. The electric and magnetic traits of ferrites are highly influenced by a variety of factors, such as temperature, processing conditions, sintering time, and chemical composition [15,16]. Spinel ferrites are mainly used in high-frequency devices [17], microwave devices [18], cancer diagnosis [19], as adsorbents in pollutant removal [20], drug delivery [21,22,23], gas sensors [24,25], and rechargeable lithium batteries [26].

Cobalt ferrite (CoFe_2_O_4_) is an important hard spinel ferrite characterized by sufficient magnetic anisotropy, high mechanical hardness, and good chemical stability [27]. Because of its diverse functional features, CoFe_2_O_4_ has attracted much interest from researchers and has numerous applications, such as drug delivery, recording media, and electromagnetic wave absorbers [28,29]. Nickel ferrites (NiFe_2_O_4_) on the other hand are mostly used as ferrofluids in high-density recording coils, magnet cores, and transformers because of their cubic spinel structure and soft magnetic materials [30,31]. Researchers have tried a variety of approaches to improve the characteristics of ferrite, including doping of RE metals and modifying crystallization techniques [32]. This led to improvement in the mechanical, electrical, structural, and magnetic behaviours of ferrites [33,34]. A study has shown that doping a low RE concertation in Ni-ferrites lowers the dielectric loss while increasing the resistivity, thus they are an ideal candidate for transformer cores [35].

Furthermore, Ga ions have been considered to significantly enhance the local crystal structure, electrical characteristics, and magnetic features of Ni–Cr ferrites with spinel structures [36]. Heiba et al. [37] employed the sol-gel method to synthesized nanocrystalline Ga-substituted Ni spinel ferrite; the product was reported to have exceptional magneto-dielectric properties. Almessiere et al. [38] used a facile hydrothermal route to produce Ni–Co SFMCs with co-doping of Ce–Dy. The magnetic, optical, dielectric, and structural characteristics were studied. Similarly, Chen et al. [39] synthesized M_x_Fe_3−x_O_4_ microspheres (M = Fe, Ni, Zn Mg, Cu, Co, Mn) for microwave heating and magnetic targeting to control drug delivery.

Several synthetic approaches have been used for the fabrication of spinel and microsphere ferrites, such as hydrothermal, spraying, gas-phase diffusion emulsion, dripping, and aerosol-assisted self-assembly methods. However, the hydrothermal method is the most effective and economical for the synthesis of ferrite microsphere [40,41]. It is a well-known fact that the hollow sphere spinel structures in the micron or submicron dimension demonstrate a superior property over their solid particle counterparts. In addition to their hollow structure and nano-scaled walls, hollow sphere spinel also tends to exhibit low density and wider surface areas which are essential in adsorption, catalytic, and gas sensing applications [42] A wide range of studies have been conducted using ferrite spheres in lithium-ion batteries, biotechnology, drugs, and photocatalysis [43,44].

This study explored the synthesis of hollow CoNiGa-SFMCs using hollow carbon spheres as templates. We then explored the effects of Ga^3+^ concentration on optical, structural, magnetic, and textural characteristics of Ni–Co SFMCs by various microscopy and spectroscopic techniques.

## 2. Experimental

For the synthesis of carbon spheres from glucose, a hydrothermal method was applied by dissolving 15 g of glucose in 85 mL at 40 °C. The solution was then transported to an autoclave in an oven at 180 °C for 12 h. The pressure inside the autoclave was initiated by carbonization. The black product was then cleaned with a water-ethanol solution and dried in an oven at 80 °C for 8 h. To prepare CoNiGa-SFMCs, required amounts of hydrates of Fe(NO_3_)_2_, Co(NO_3_)_2_, Ga(NO_3_)_2_, and Ni(NO_3_)_2_ were dissolved in 20 mL deionized (DI) water at an ambient temperature. Next, 100 mg of carbon spheres, 1.2 g of urea, and 1.2 g of ascorbic acid were added to 30 mL DI H_2_O, mixed until the solution turned clear, and the mixture was sonicated for 30 min. The solid product was poured into a stainless-steel autoclave, followed by a heat treatment for 12 h at 180 °C. CoNiGa-SFMCs were washed with hot DI water, filtered, and dried in an oven.

The crystal structure was investigated using a Rigaku Benchtop Miniflex X-ray diffractometer (XRD) (λ = 1.54059 Å). The morphology was investigated by scanning electron microscopy (SEM), transmission electron microscopy (TEM) using Titan-FEI-Morgagni-268, and elemental analysis/mapping was performed using its attached EDX module. ASAP-2020 Plus (Micromeritics, Norcross, GA, USA) was used to measure the texture properties of the products. Surface areas were obtained using the Brunauer-Emmett-Teller (BET) equation. Diffuse reflectance (DR) analysis was performed using a UV-vis spectrophotometer, which is the standard method for determining the absorption properties of powder specimens. Magnetization results were acquired from a VSM-coupled PPMS (Quantum Design PPMS DynaCool-9).

## 3. Results and Discussions

### 3.1. Microstructural Features

**Figure 1:**
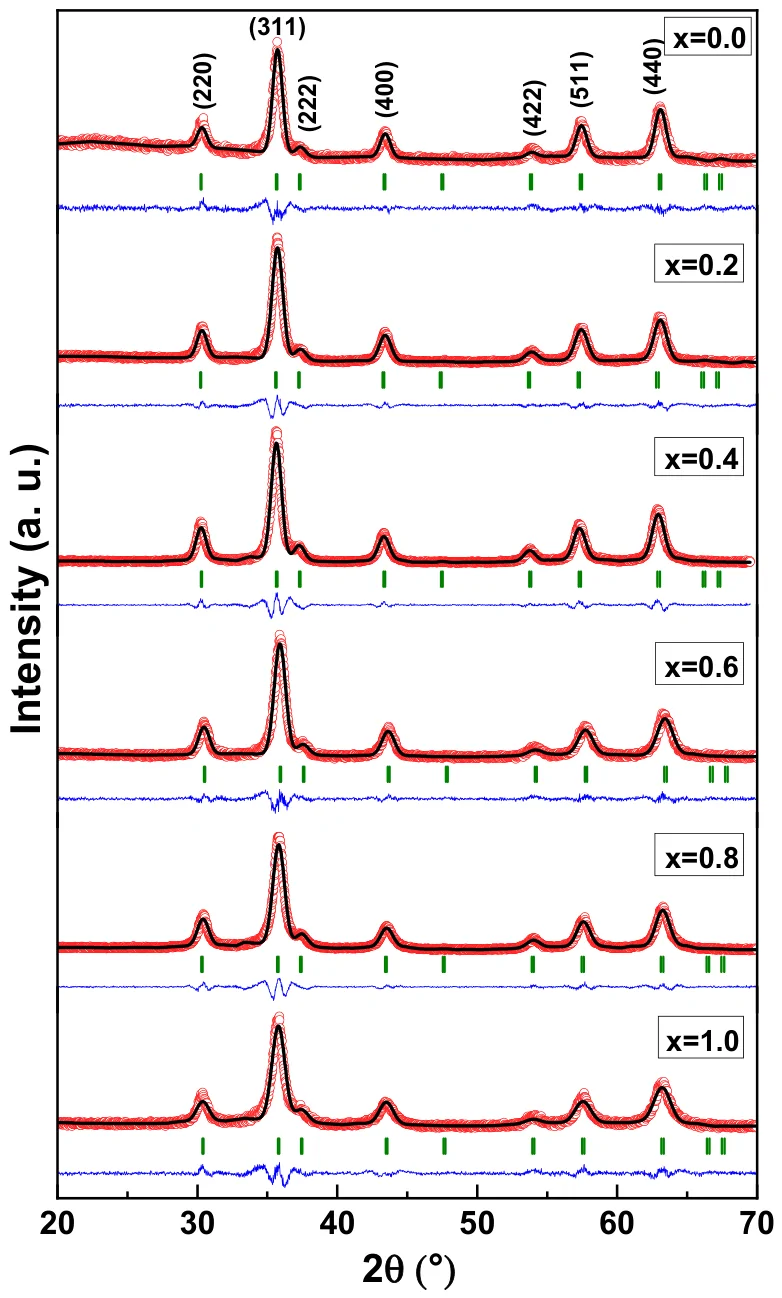
CoNiGa-SFMC XRD powder diffraction patterns. Red dots, black solid lines, blue solid lines, and green bars represent the experimental data, calculated refinement models, difference profiles, and Bragg positions, respectively.

Figure 1 shows the X-ray powder diffraction patterns of CoNiGa-SFMCs. The XRD patterns of all the products exhibited a typical phase of cubic spinel ferrite according to card number JCPDS 10-0325. This can be evidenced by the diffraction lines with the following hkl values (220), (311), (222), (400), (422), (511), and (440). No extra peaks for the second phase or carbon in the composites were observed, thus indicating the effectiveness of the hydrothermal method currently employed in the preparation of the SFMCs. It is interesting to note the broadening of the diffraction lines (Figure 1), which, according to the theory of kinematical scattering, can be ascribed to small crystal formation in the nano dimension. The crystal size (D_XRD_), cell parameters, and cell volume were measured by refinement using Match3! and are presented in Table 1. Increasing the Ga concentration from x = 0.00 to 0.4 increased the cell parameters because of the expansion of the spinel crystal (mainly owing to the difference in r_Ga_^3+^ and r_Fe_^3+^). The cell parameters at x = 0.06 decreased, then slightly increased at x = 0.08 and 0.10; this can be explained by the presence of the Ga^3+^ ion at the grain interfaces, which causes internal stress when the substituent concentration increases, the grain growth at the crystal lattice sites becomes restricted, and consequently, the crystal size decreases [45,46]. The average crystal size was calculated using the Scherrer Equation, D_XRD_ = Kλ/(βCosθ), the most intense peak (311) was found between 9 and 12 nm.

**Figure 2:**
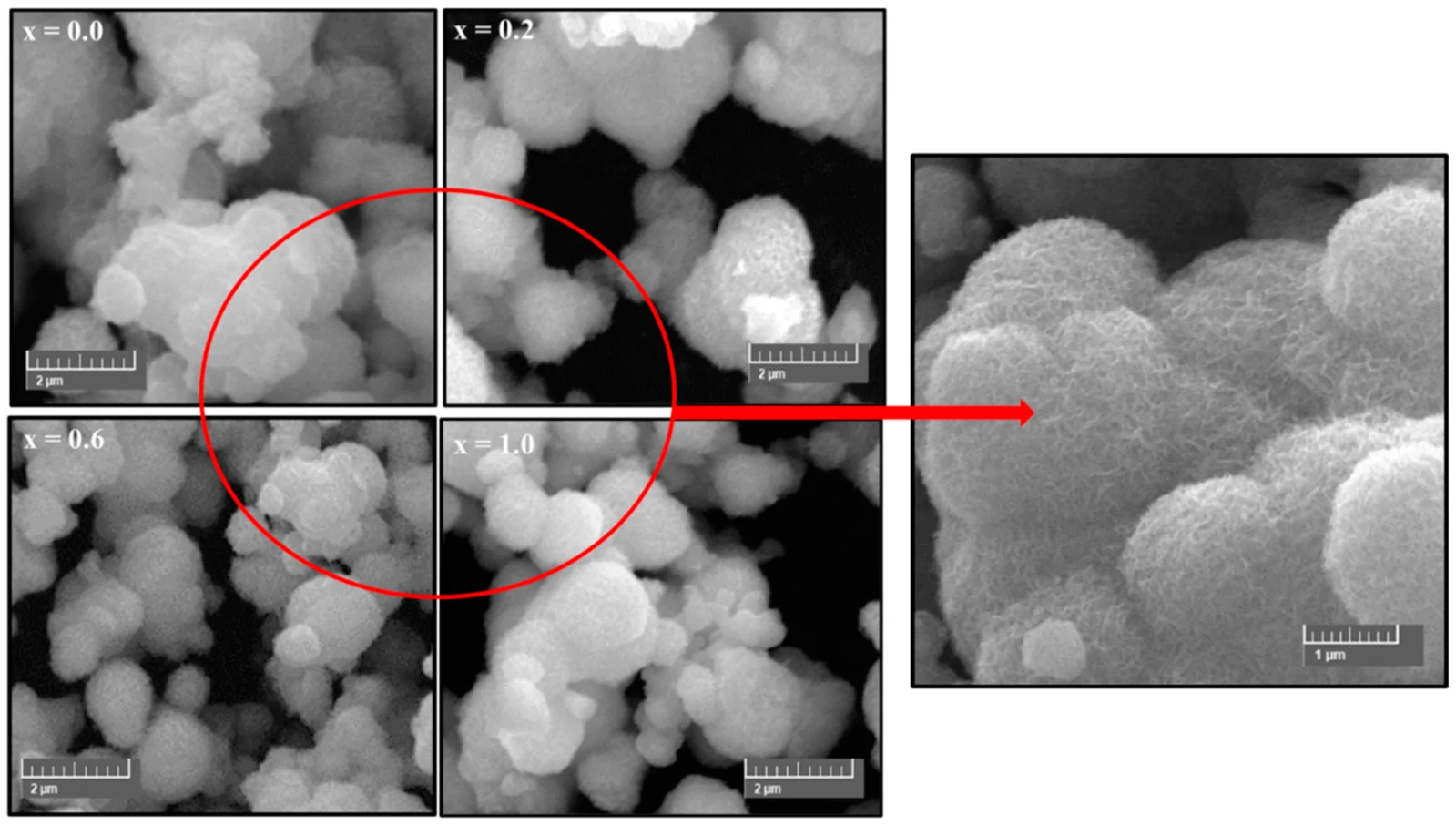
CoNiGa-SFMCs’ SEM images.

Figure 2 shows a morphological investigation of the CoNiGa-SFMCs for x = 0.0, 0.2, 0.6, and 1.0.

**Figure 3:**
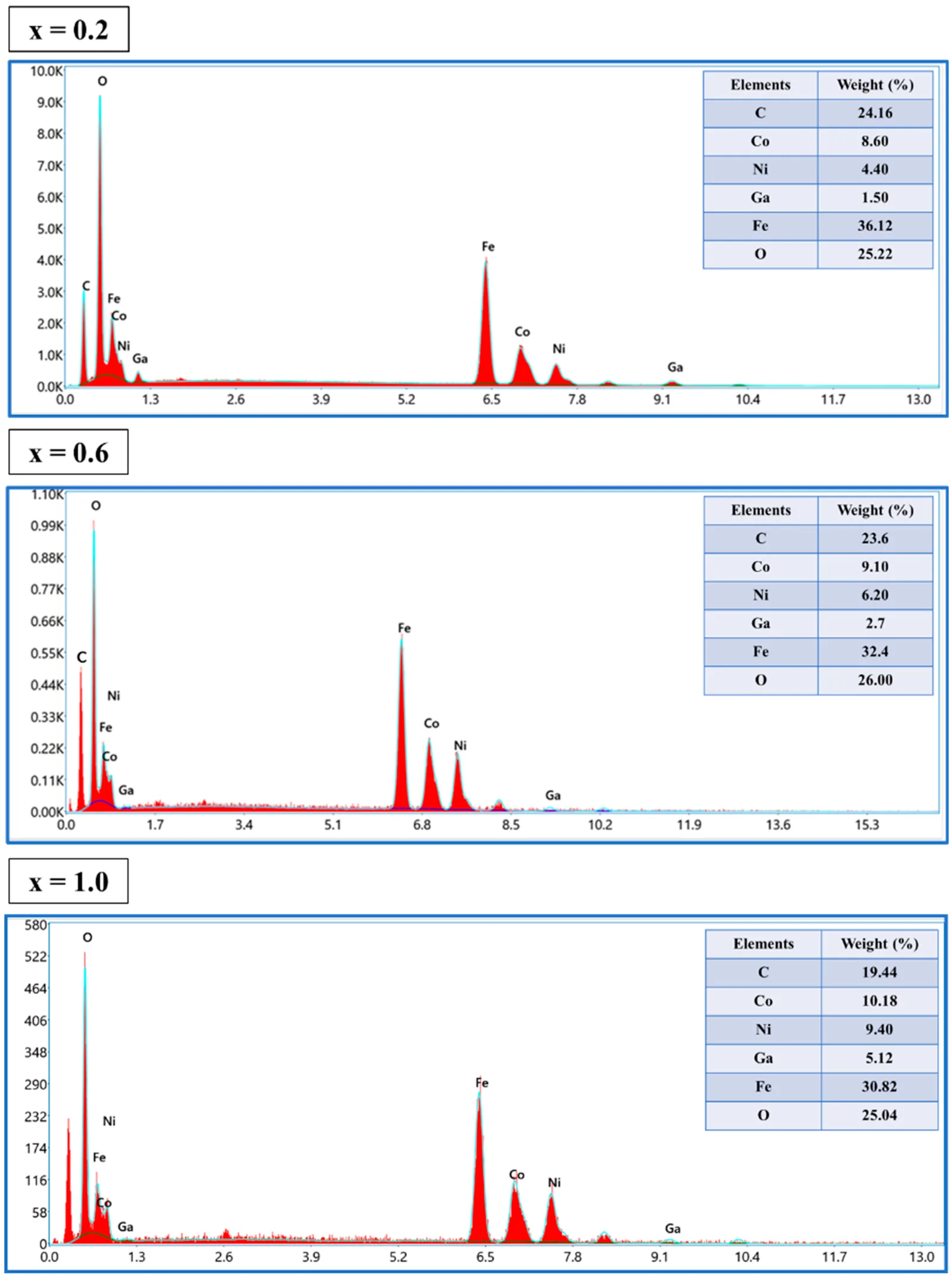
EDX spectra of CoNiGa-SFMCs (x = 0.2, 0.6, and 1.0) SFMCs.

**Figure 4:**
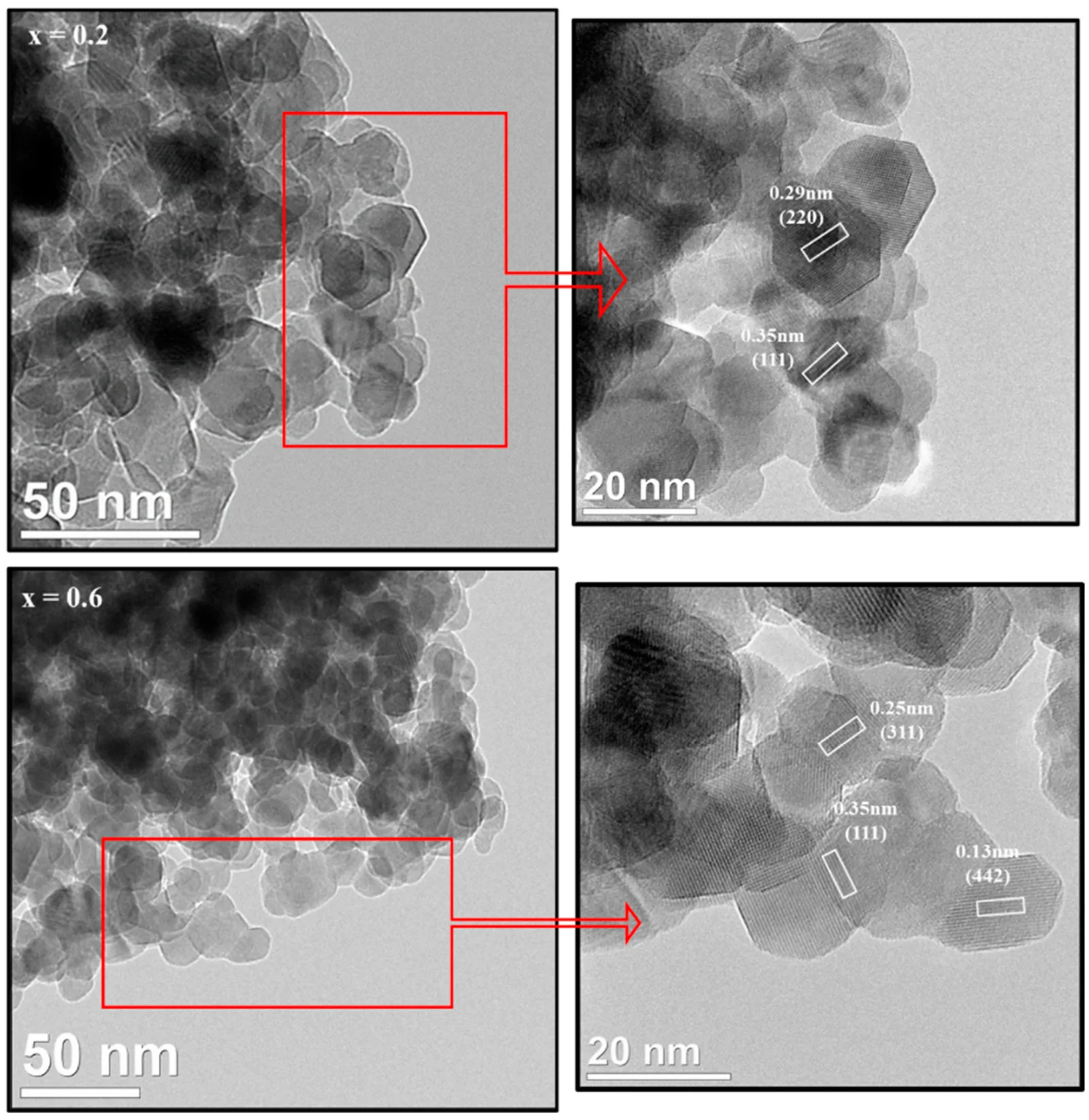
TEM and HR-TEM micrographs for CoNiGa-SFMCs for x = 0.2 and 0.6.

The images clearly show aggregated particles of microspheres with different diameters. The microspheres had a rough surface with a diameter of approximately 1 µm. The EDX spectra represented the elemental analysis of selective ratios of CoNiGa-SFMCs (x = 0.2, 0.6 and 1.0). All spectrums exhibited the following elements: Ni, Co, Fe, Ga, C, and O, which approved the chemical composition without any impurities, as displayed in Figure 3. In addition, TEM images of CoNiGa-SFMCs (x = 0.2 and 0.6) confirmed the presence of spherical particles in Figure 4. HR-TEM images showed the spinel structure by evaluating the lattice planer using ImageJ software as follows: 0.13, 0.25, 0.29, and 0.35 nm, which belong to the Miller indexes (422), (311), (220), and (111), respectively.

### 3.2. Nitrogen Physisorption

**Figure 5:**
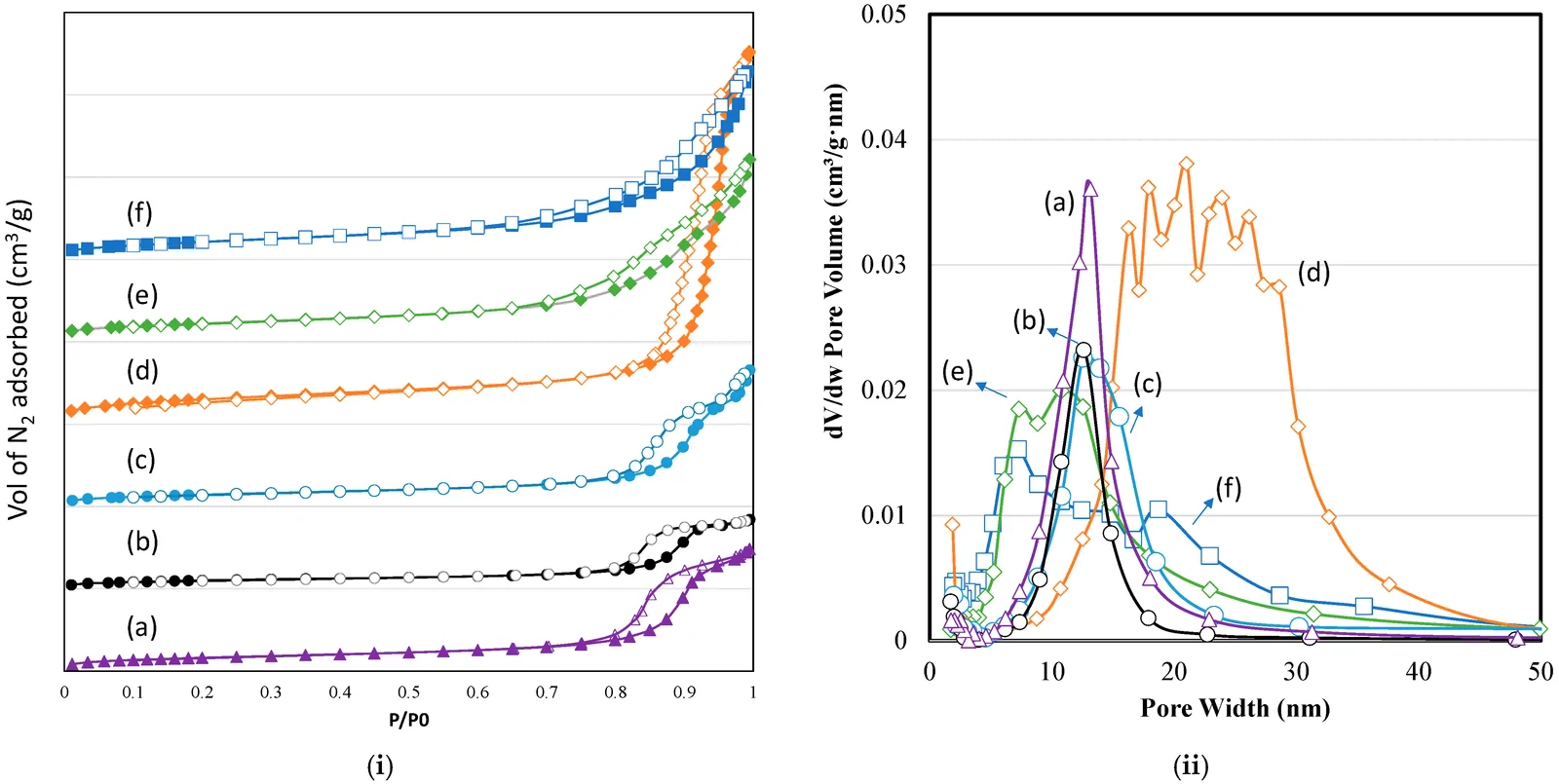
Surface area measurement using N_2_ adsorption isotherm (**i**) and its derivative (**ii**) of CoNiGa-SFMCs where x is (a) 0.0, (b) 0.2, (c) 0.4, (d) 0.6, (e) 0.8, and (f) 1.0.

Differential hysteresis loops which carried out adsorption–desorption isotherms were obtained from N_2_ physisorption at −196 °C. The BET equation was applied to calculate surface areas of all samples [47]. A mesostructure of variable quality was observed at a relative pressure between 0.8 and 1.0. Figure 5 shows the isotherm patterns and pore size distributions of the CoNiGa-SFMCs.

Table 2 presents textural characteristics, such as pore size distributions, pore volume, and surface area. CoNiGa-SFMCs for x = 0.0, 0.2, and 0.4 showed an H3 hysteresis loop is present in a type-IV isotherm, indicating the formation of mesopores. The CoNiGa-SFMCs (x = 0.0) exhibited a surface area of 57 m^2^∙g^−1^.

After the addition of Ga (x = 0.2), a slight reduction in surface area was observed (36 m^2^∙g^−1^). Similarly, the pore volume was reduced from 0.22 cm^3^∙g^−1^ to 0.13 cm^3^∙g^−1^ with the addition of Ga. This pattern indicates external imperforation with the addition of Ga to Ni–Co ferrite; however, the textural features recovered with further Ga doping (x = 0.4–1.0). The pore size distribution curve revealed the presence of unimodal pores centered at approximately 13.0 nm. The Ga doping reached x = 0.6, and the sample isotherm indicated a type-IV isotherm; however, the hysteresis loops changed to H1, which indicated a unique mesostructure compared to that at x ≤ 0.4. The surface area increased to a maximum of 105 m^2^∙g^−1^ and a 0.68 cm^3^∙g^−1^ pore volume. The pore size distribution curve shows the presence of multimodal porosity, and the average pore size distribution increased to 26.2 nm. Such an increase in textural characteristics is mainly attributed to defects and vacancies in a spinel structure [48].

At the same time, when the Ga doping was increased to x = 0.8, a curvature-type hysteresis was observed, indicating bimodal pores. The pore size distribution curve showed two major peaks at approximately 7.3 and 10.9 nm with an average pore size centered at approximately 14.2 nm. The CoNiGa-SFMCs (x = 1.0) showed similar hysteresis, with two types of pores centered between 6 and 19 nm. Hai et al. [49] reported that Co_0.5_Ni_0.5_Fe_2_O_4_ with a mesostructure can be obtained with an estimated surface area between 8 and 24 m^2^∙g^−1^. The presence of a high surface area of up to 105 m^2^∙g^−1^ in this study indicates that Ga doping in Co–Ni ferrite increases the surface area and generates multimodal pores.

### 3.3. Optical Properties

**Figure 6:**
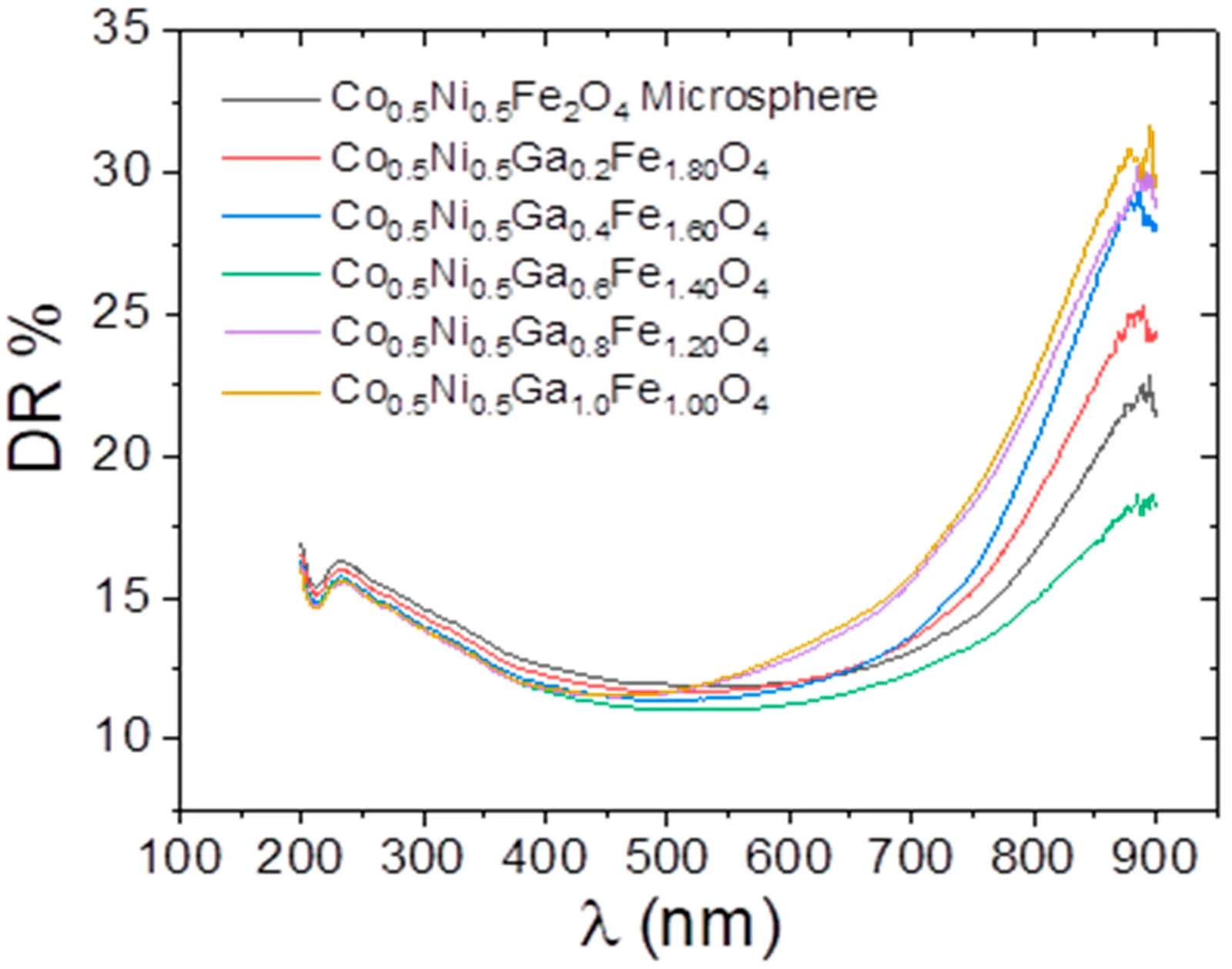
Percent diffuse reflectance spectra of CoNiGa-SFMCs.

Diffuse reflectance (DR) analysis was performed using a UV-vis spectrophotometer, which is the standard method for determining the absorption properties of powder specimens. Figure 6 shows the %DR (200–900) spectra of CoNiGa-SFMCs. Mixed spinel Co_0.5_Ni_0.5_Fe_2_O_4_ SFMCs had reflectance intensities varying in a narrow range between 11.92% and 22.76% over the entire sweep range of the incident beam. However, the reflectance intensities of the Ga^3+^-substituted samples differed significantly above 500 nm. Among them, Co_0.5_Ni_0.5_Ga_1.0_Fe_1.0_O_4_ SFMs had the widest intensity range between 11.50% and 31.50% in the 500–900-nm wavelength region.

In addition to the optical absorption properties, some optoelectronic properties, such as *E*_g_ or band gap, can be estimated using DR data. The evaluation process can be explained by the Kubelka–Munk (K–M) theory, which assigns a reflectance ( $R_{\infty }$ )-dependent function $F\left(R_{\infty }\right)$ related with *α* (optical absorption coefficient) as [50]:

(1) $$F\left(R_{\infty }\right)=\frac{{\left(1-R_{\infty }\right)}^{2}}{2R_{\infty }}=\frac{K}{S}=\alpha ;$$

where *K* is the K–M absorption and scattering coefficient, *S* is the K–M scattering coefficient, and ∞ defines a sufficiently thick layer that typically requires a sample depth of 1–3 mm to scatter or absorb all the incident radiation [51].

Considering the peculiarities of the band structure theory for direct band gap semiconducting materials, the linear relation between *E*_g_ and α is [52]:

(2) $$\alpha .h\upsilon =A_{1}{\left(h\upsilon -E_{g}\right)}^{1/2};$$

where $A_{1}$ is an arbitrary proportionality constant and hυ is the incident light energy. We can plot a graph of ${\left(F\left(R_{\infty }\right)h\upsilon \right)}^{2}$ by replacing α with $F\left(R_{\infty }\right)$ in Equation (2) and taking square of both sides:

(3) $${\left(F\left(R_{\infty }\right)\cdot h\upsilon \right)}^{2}=A_{2}\left(h\upsilon -E_{g}\right);$$

**Figure 7:**
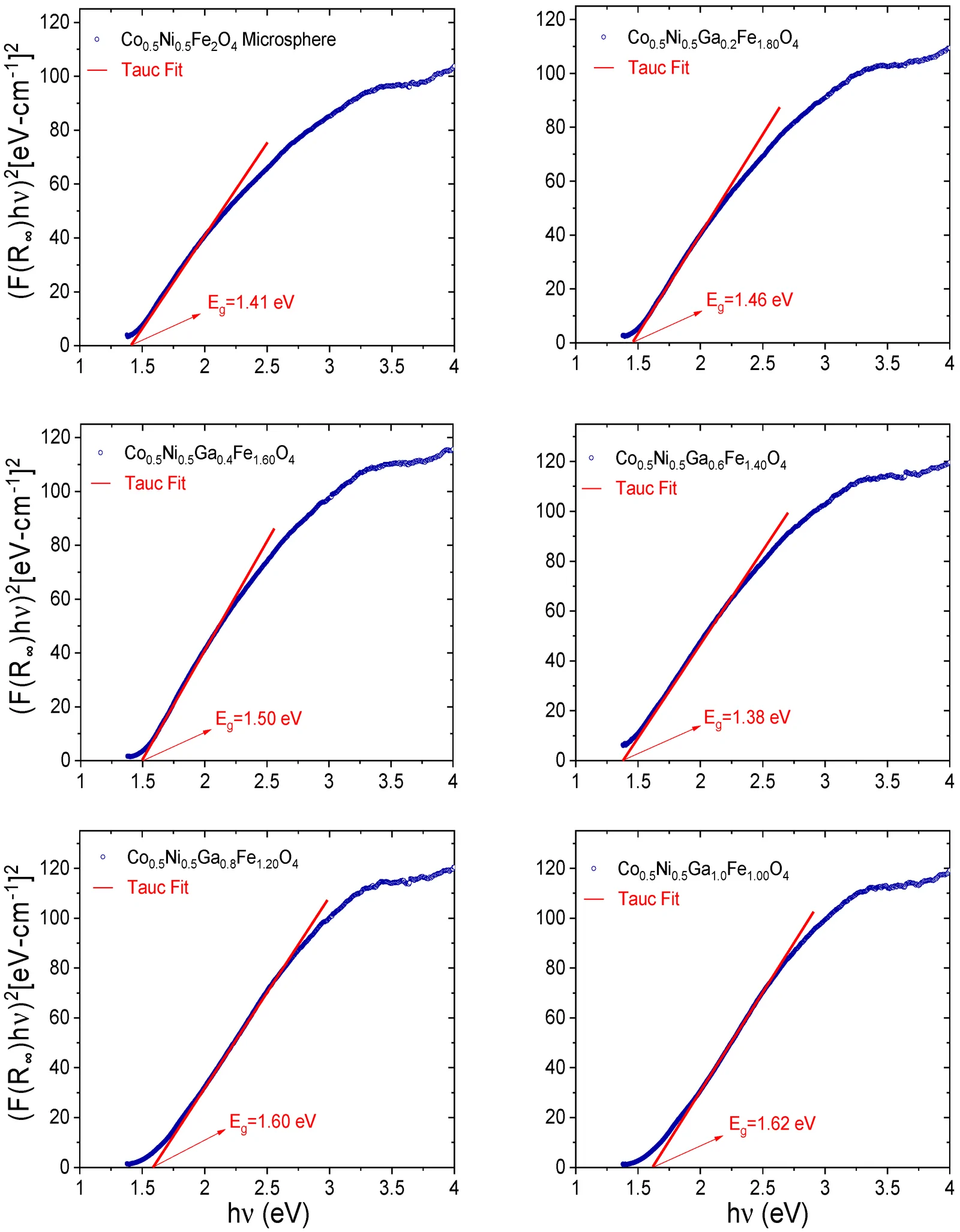
Tauc plots and estimated optical energy band gaps of CoNiGa-SFMCs.

The values of *E*_g_ can be extracted by plotting ${\left(F\left(R_{\infty }\right)h\upsilon \right)}^{2}$ versus hυ graphs. The crossing point of the linear fit at the rising linear part and abscissa immediately yields *E*_g_ in eV units. This type of procedure for the quantitative determination of optical energy band gaps is known as a Tauc plot [53,54,55] as presented in Figure 7.

Figure 7 shows all the Tauc plots and estimated band gaps of the microspheres. The pristine Co_0.5_Ni_0.5_Fe_2_O_4_ SFMs had a gap of 1.41 eV, while the Ga^3+^-ion substituted samples had band gap magnitudes of 1.46, 1.50, 1.38, 1.60, and 1.62 eV, corresponding to an increase in Ga^3+^ concentrations of x = 0.2 to 1.00.

When the minimum magnitudes of 1.38 eV data belonging to the Co_0.5_Ni_0.5_Ga_0.6_Fe_1.4_O_4_ SFMC wwere excluded, the substitution sprocess of Ga^3+^ increased the *E*_g_ magnitude of the pristine specimens almost linearly. Furthermore, all microsphere samples synthesized by the sol-gel method had *E*_g_ values in the order of those of semiconductor materials. Some researchers have reported that 1.53 eV of direct and 1.625 eV of indirect *E*_g_ values for mixed Co_0.5_Ni_0.5_Fe_2_O_4_ SFMCs obtained via low-temperature solution combustion and hydrothermal processes, respectively [56,57].

### 3.4. Magnetic Features

**Figure 8:**
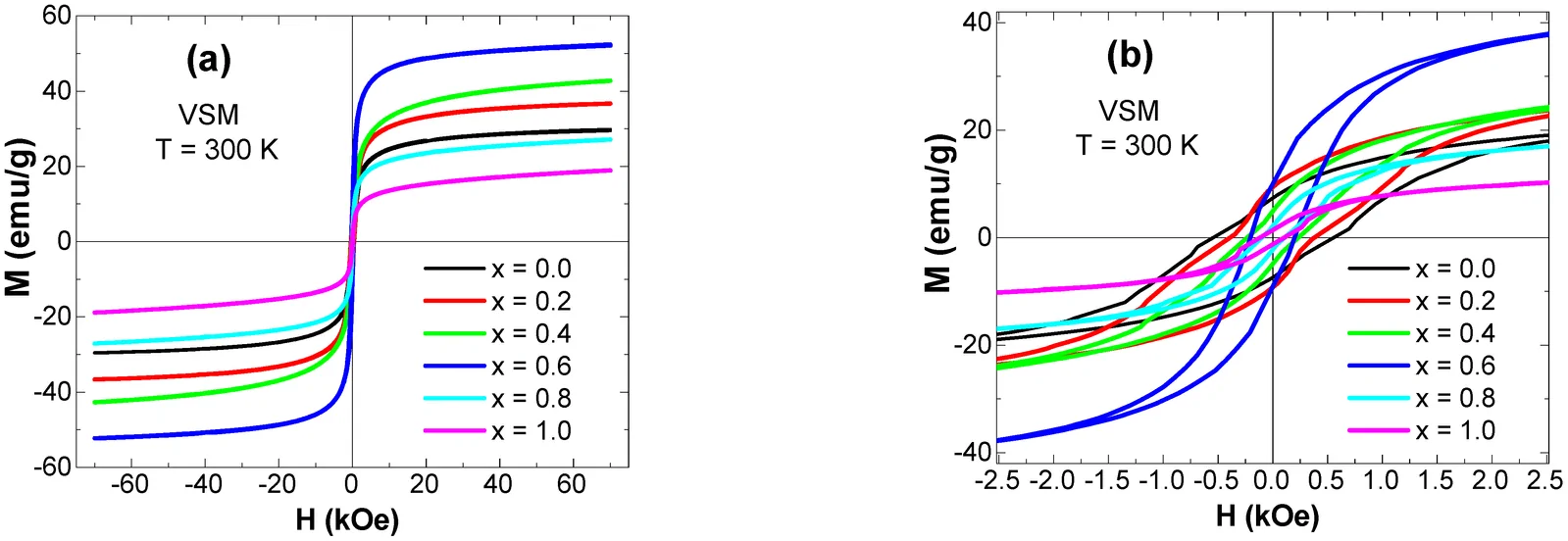
Variation of magnetization with respect to applied magnetic field (M–H) at 300 K for CoNiGa-SFMCs in different scales (**a**,**b**).

**Figure 9:**
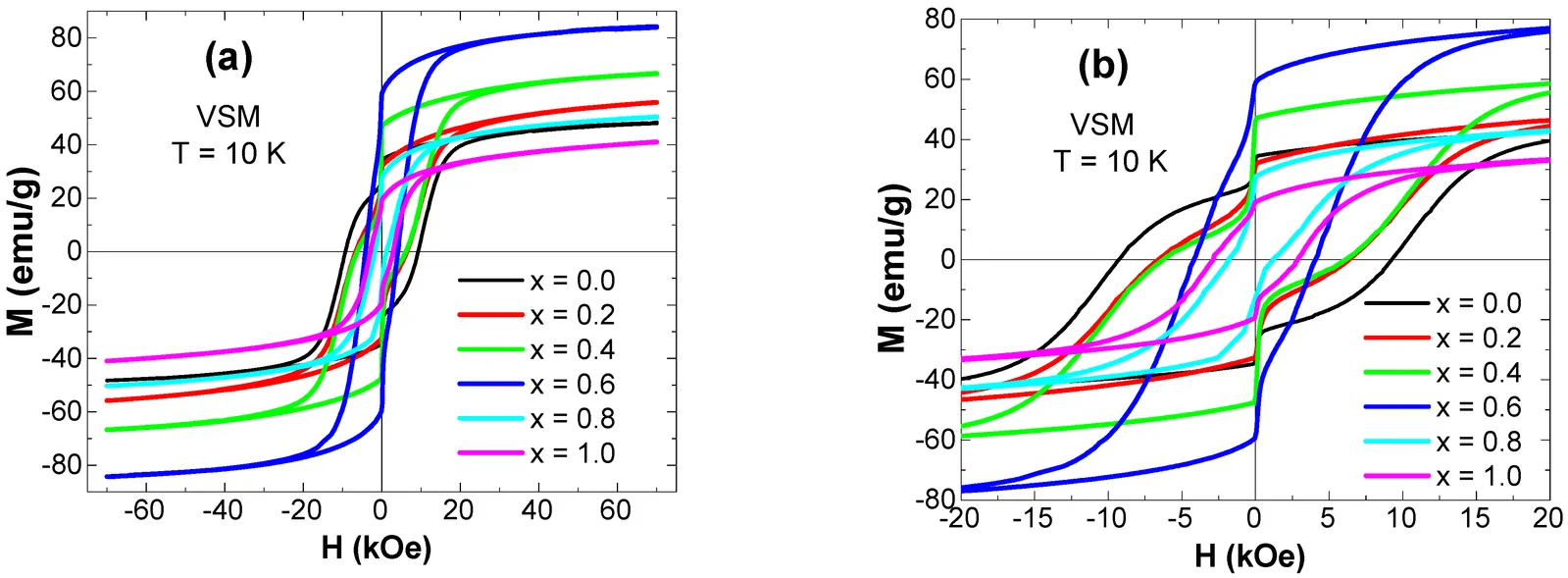
M–H curves at 10 K for different CoNiGa-SFMCs in different scales (**a**,**b**).

The evolution of magnetization with respect to the external magnetic field (M–H) at room (300 K) and low (10 K) temperatures are illustrated in Figure 8 and Figure 9, respectively, for all CoNiGa-SFMCs.

The M–H measurements were performed in a wide magnetic field (70 kOe). The enlarged views of the M-H curves indicate that the different ferrite microspheres exhibited clear hysteretic behavior. At 300 K, the diapason M_s_, M_r_, and H_c_ were 19.0–52.4 emu/g, 2.1–10.1 emu/g, and 98–545 Oe, respectively. M_s_, M_r_, and H_c_ values at 10 K were in the ranges 41.0–84.3 emu/g, 22.2–59.0 emu/g, and 1804–9298 Oe, respectively.

Accordingly, the recorded M–H curves were typical of samples displaying ferrimagnetic ordering, where clear loops with hysteresis-like behavior (with no negligible coercivity and remanence) were observed for all compositions. Compared with those registered at 300 K, the M_s_, M_r_, and H_c_ data observed at 10 K increased significantly. M_s_ and M_r_ values at 10 K were at least 1.5–2 times and 6–14 times greater, respectively, with respect to those at 300 K. H_c_ values at 10 K were at least 16 times greater and even more with respect to those at 300 K. The enhancement in the magnetic parameters can be ascribed to the reduction in thermal fluctuations owing to temperature reduction [58,59].

**Figure 10:**
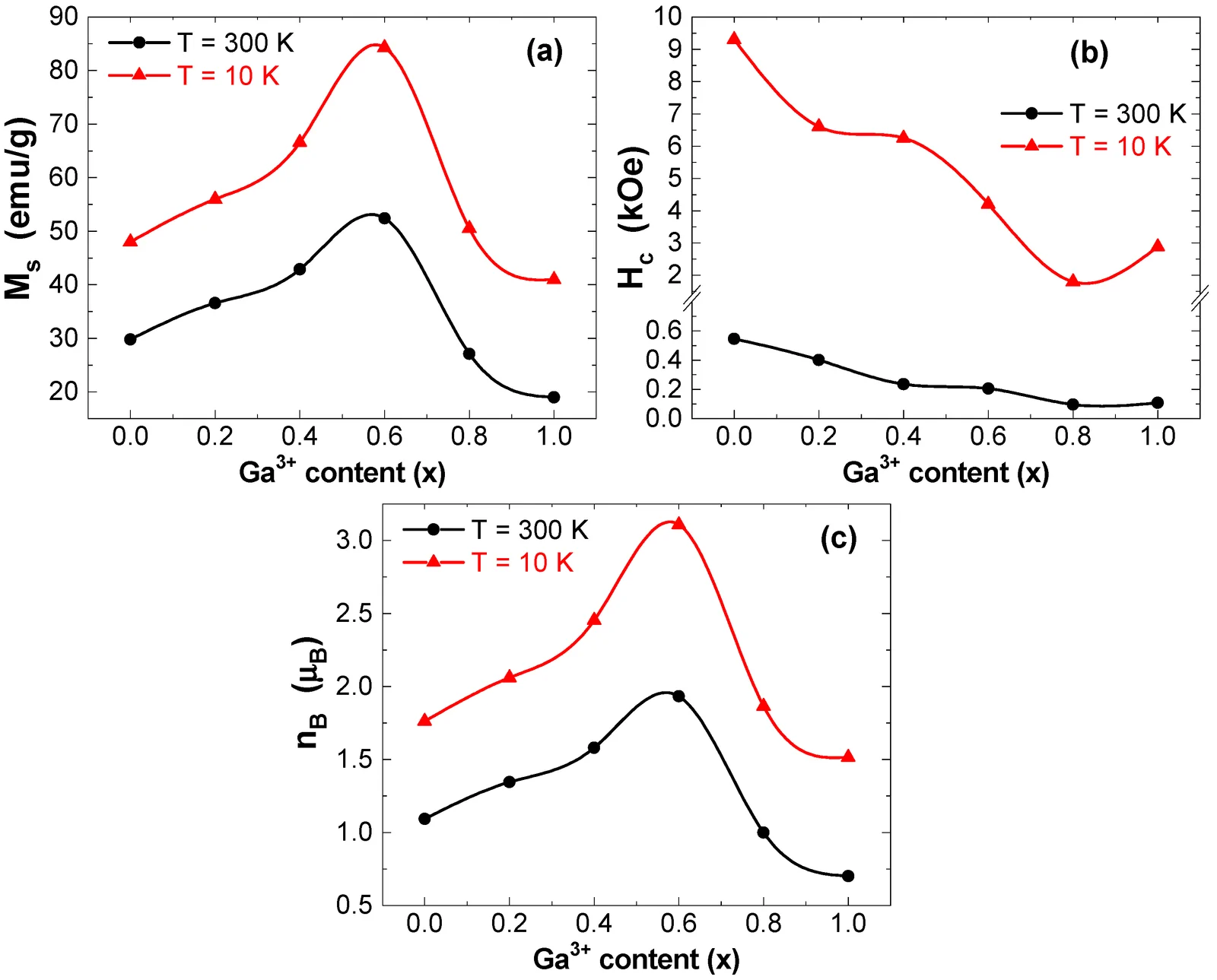
Concentration dependences of main magnetic characteristics: (**a**) saturation magnetizations (M_s_), (**b**) coercive fields (H_c_), and (**c**) magnetic moments ( $n_{B}$ ) for CoNiGa-SFMCs with respect to the concentration of Ga^3+^ ions.

The M–H results revealed significant variations in the magnetic parameters of CoNiGa-SFMCs, suggesting the inclusion of Ga ions into the Co–Ni ferrite. Figure 10 presents the variations in saturation magnetizations (M_s_), coercive fields (H_c_), and magnetic moments ( $n_{B}$ ) extracted from the M-H hysteresis loop measurements with respect to the concentration of Ga^3+^ ions.

Other parameters, such as the remanence (M_r_) and squareness ratio (SR, which is the ratio of M_r_/M_s_) are listed in Table 3. The M_s_ values obtained for the nanocrystalline samples were noticeably lower than those of the bulk material. The surface areas became larger for nanosized ferrite particles, hence the surface tension and surface energy also increased. These provoke variations in the sites’ preferences for cations, leading to an increase in the degree of defects; consequently, a lower value of magnetization will be observed [60].

Usually, the magnetic features of ferrite nanomaterials are dictated by possible B–B, A–B, and A–A interactions and the cation allocation between the different sites [61,62]. M_s_ increased gradually with a rise of Ga^3+^ ion content till x reached 0.6, followed by a progressive drop with a much higher x content. The maximum M_s_ values obtained in the microsphere sample with an x concentration of 0.6 (i.e., Co_0.5_Ni_0.5_Ga_0.6_Fe_1.4_O_4_) reached 52.4 and 84.3 emu/g at 300 and 10 K, respectively. However, the lowest M_s_ values obtained for the Co_0.5_Ni_0.5_Ga_1.0_Fe_1.0_O_4_ microsphere sample (x = 1.0) were approximately 19.0 and 41.0 emu/g at 300 and 10 K, respectively. Similarly, the maximum M_r_ values of the Co_0.5_Ni_0.5_Ga_0.6_Fe_1.4_O_4_ microsphere sample were 10.1 and 59.0 emu/g at 300 and 10 K, respectively.

The decrease in M_s_ values can be debated based on Neel’s theory [63,64,65]. Based on this model, the entire magnetization is the difference in magnetic moments between the B and A sites. In ferromagnetic nanomaterials, if the magnetic moments of the B and A sites are M_B_ and M_A_, respectively, the entire magnetic moment M = M_B_ − M_A_. In the CoNiGa-SFMCs, the magnetic moments of the constituent ions were approximately Co^2+^ = 3 μ_B_, Ni^2+^ = 2.8 μ_B_, Ga^3+^ = 3 μ_B_, and Fe^3+^ = 5 μ_B_.

Usually, Ni spinel ferrite nanoparticles have an inverse spinel structure, where Ni^2+^ ions reside in O′ sites, whereas Fe^3+^ ions occupy both the A and B sites [66]. Nanoparticles of Co spinel ferrite have a mixed spinel structure, where Co^2+^ and Fe^3+^ ions reside at the T_d_ and O_h_ sites [67]. The introduction of Ga^3+^ ions to substitute Fe^3+^ ions caused a change in the allocation of ions between the A and B sites.

We found that the CoNiGa-SFMCs were neither completely inverse nor normal. The Ga^3+^ ions entered and mainly accumulated at the A sites at lower x values, whereas they mainly accumulated at the B site for higher x values [66,68]. Therefore, for x contents below 0.6, higher amounts of Ga^3+^ ions replace the Fe^3+^ ions residing in the A site. This provokes a weakening in superexchange interactions among the A and B sites and results in a reduction of magnetic moments of A sites, and consequently, the net magnetization increases. This could explain the continuous increase in M_s_ values at 300 and 10 K for CoNiGa-SFMCs with x ≤ 0.6. For x contents above 0.6, some Ga^3+^ ions start to substitute Fe^3+^ ions residing at B sites. When Ga^3+^ ions tend to substitute Fe^3+^ ions at B sites, Ga–Fe interactions occur, which are very weak in comparison to Fe–Fe interactions. Such an effect leads to a reduction in magnetic moments of B sites, and consequently, the net magnetization starts to decrease at higher x values. This could explain the decrease in M_s_ values at 300 and 10 K for the CoNiGa-SFMCs with x > 0.6. Thus, by adjusting the Ga^3+^ content, it is feasible to alter the magnetic features of nanomaterials for certain applications.

The observed magnetic moments ( $n_{B}$ ) per formula unit in μ_B_ (Figure 10c) were assessed using the expression [69,70]:

(4) $$n_{B}=\frac{MW\times M_{s}}{5585};$$

where MW represents the molecular weight of the sample. The pristine Co–Ni spinel ferrite microspheres exhibited $n_{B}$ values of approximately 1.09 and 1.79 μ_B_ at 300 and 10 K, correspondingly.

The $n_{B}$ values increased gradually in parallel with the ratio of Ga^3+^ ions up to x = 0.6. The maximum $n_{B}$ values for this composition were 1.93 and 3.11 μ_B_ at 300 and 10 K, respectively. Then, the $n_{B}$ values decreased progressively for a much higher x content. The lowest $n_{B}$ values for the x = 1.0 composition were 0.70 and 1.52 μ_B_ at 300 and 10 K, respectively. The $n_{B}$ values were in good agreement with the M_s_ values, which could explain the variation in magnetization with Ga substitution.

The changes in the M_s_ values can also be related to the evolution of crystallites/particle size. Generally, M_s_ decreases with a reduction in the crystallite/particle size and vice versa. In this investigation, the crystallite/particle size increased gradually with an increase in the content of Ga^3+^ ions up to x = 0.6 and then decreased slightly for much higher x concentrations. This is consistent with the M_s_ values at both 300 and 10 K.

The changes in the H_c_ data versus the Ga concentration (x) are presented in Figure 9b. The coercive fields (H_c_) of CoNiGa-SFMCs diminished with an increase in the amount of Ga^3+^ ions, showing that the Co–Ni SFMCs were softer (in terms of coercivity) with Ga substitution. This could be ascribed to the small number of pores within the structure with small crystal grains [71]. Hence, the displacement of magnetic domain walls was easier, and consequently, lower H_c_ values could be achieved [72]. Furthermore, the reduction in H_c_ values with an increase in the Ga content can be ascribed to the decline in anisotropy fields, which in turn reduces the domain wall energy [73,74]. The reduction in the crystallites/particle size upon Ga substitution also explains the decline in the H_c_ values.

Using the H_c_ and M_s_ values, the anisotropy constant can be determined as [75]:

(5) $$H_{c}=\frac{0.98\;K}{M_{s}};$$

The calculated K values are listed in Table 3. As shown, the K values mainly decreased with an increase in Ga substitution. In the current study, the greatest anisotropy values at both 300 and 10 K occurred at the x = 0.0 composition, while the lowest anisotropy values at both 300 and 10 K occurred at the x = 0.8 composition. Variations in *K* values may have been provoked by either of the following mechanisms [76]:

(i) as per the model of one-ion anisotropy, the high anisotropy of Co–Ni ferrites is largely owing to the existence of Co^2+^ and Ni^2+^ ions at the B site of spinel nanomaterial, and
(ii) the occurrence of Ga^3+^ ions in the A site reduces the anisotropy owing to the decrease in A–B superexchange coupling.

The anisotropy might decrease because of the additional Ga^3+^ substitutions at the B sites of the present nanomaterials and the transfer of Co^2+^ ions into the A sites. It could also decrease because of the decrease in A–B super-exchange coupling. This leads to noncollinear spin arrangements [76].

As reported previously in numerous studies, SR ratios below 0.5. infer that a nanomaterial is in the multi-magnetic domain size, while SR ratios at or above 0.5 are assigned to the forming of a single magnetic domain [58,59,77]. The obtained SR values at 300 K were below 0.5 and can be assigned to a multi-magnetic domain structure for all compositions at room temperature. However, the calculated SR values at 10 K were above 0.5, indicative of a single magnetic domain for all compositions at extremely low temperatures.

**Figure 11:**
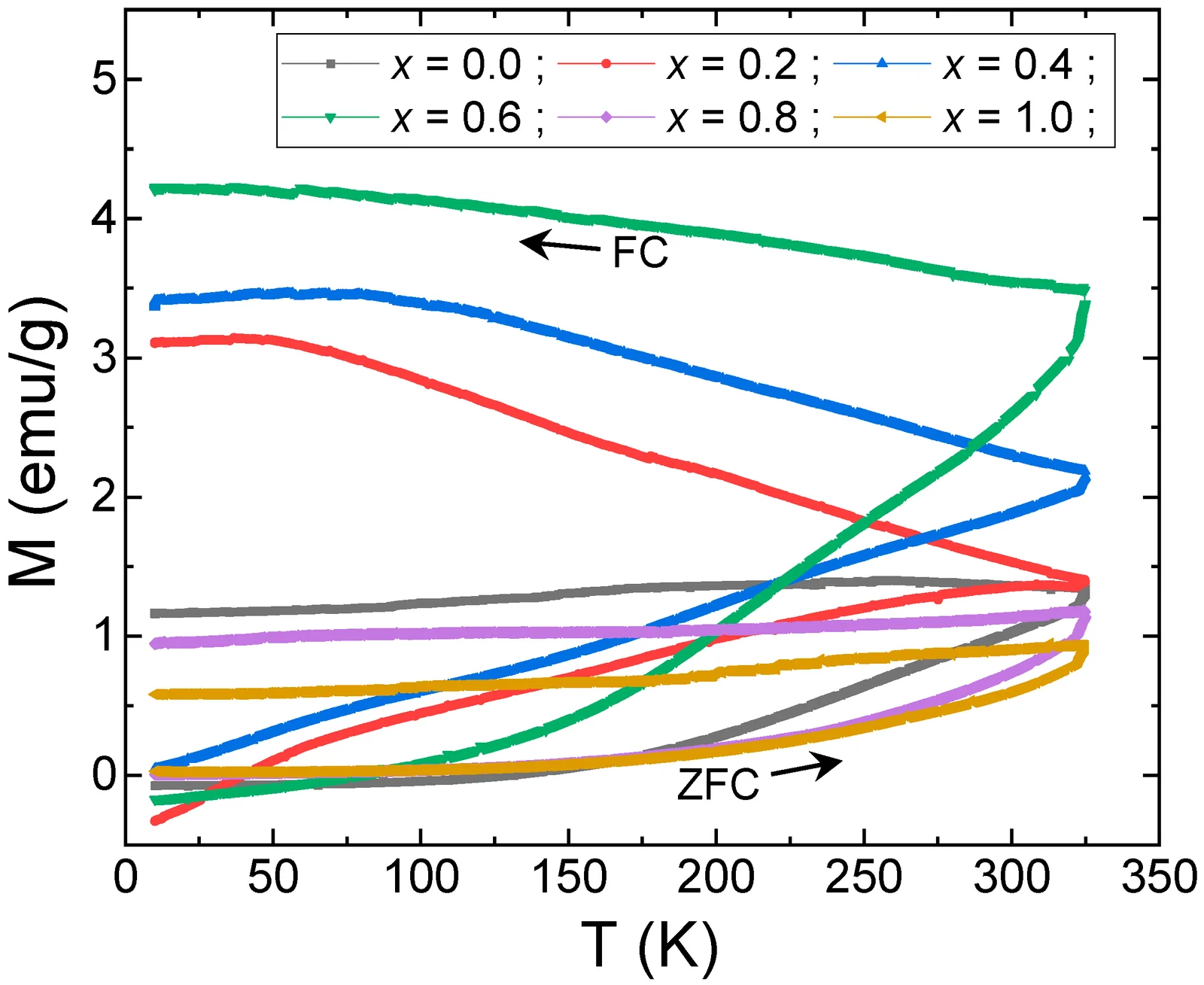
Curves of temperature-dependent magnetization performed under zero-field cooling (ZFC) and field-cooled (FC) conditions for CoNiGa-SFMCs.

Temperature-dependent magnetization data were obtained under zero-field cooling (ZFC) and field-cooled (FC) conditions and presented in Figure 11. The curves of the ZFC–FC magnetizations were acquired using a previously described procedure [78,79]. First, the sample was cooled to 10 K under a minimal zero field. Subsequently, a field of approximately 100 Oe was applied to the sample. ZFC magnetization data were recorded as the product was heated to approximately 320 K. However, the FC magnetization data were compiled as the product was cooled again to 10 K (at the same applied field of 100 Oe).

Figure 11 presents the ZFC–FC magnetizations for all CoNiGa-SFMCs. A large separation between the FC and ZFC curves was observed for all prepared CoNiGa-SFMCs. The amplitude of the FC magnetization portion increased gradually with an increase in the Ga^3+^ concentration up to x = 0.6, and then decreased progressively for a much higher x. This tendency was consistent with that observed for M_s_ versus Ga^3+^ doping.

According to Figure 11, the pristine and CoNiGa-SFMCs were in the ferromagnetic phase over the entire temperature range. Usually, a peak in the ZFC curve denotes the mean blocking temperature (T_B_) [80,81]. In the present case, no peak was observed in any of the ZFC curves, indicating that the T_B_ did not appear and was much higher than the maximum temperature measured (i.e., T_B_ exceeded 320 K). Because of the device temperature range limits, the ZFC–FC measurements could not be performed at much higher temperatures. These results were in line with the equivalent M-H data obtained at 300 and 10 K for all CoNiGa-SFMCs and confirms the ferromagnetic behavior of all samples.

The curves of the ZFC–FC magnetizations of various prepared ferrites can be understood according to the following descriptions. Once SFMs are cooled without a magnetic field, their magnetization aligns towards the easy axis and cannot be switched further due to a magnetic anisotropy energy barrier which rises as the temperature drops. Because the orientations of the easy axes of the NPs are arbitrary, when the temperature becomes extremely low, the global magnetization approaches zero. At this moment, once the NPs are heated to 320 K under an applied magnetic field, a certain amount of thermal energy will be gained by the NPs, which may be enough to change the magnetization of the NPs from the easy axes toward the direction of the field. Thus, the global magnetization of the product increases as revealed in all the ZFC portions of Figure 11.

For FC magnetization measurements, usually, when the temperature is reduced, the magnetic moments of the NPs tend to be aligned with the easy axes nearest to the direction of the field and remain locked in that direction. As shown in Figure 11, the behavior of the FC magnetization curves was relatively flat over the entire temperature range (cases x = 0.0, 0.8, and 1.0) or showed a continuous growth with a reduction in temperature and then flattened (saturation) at extremely low temperatures (cases x = 0.2, 0.4, and 0.6). These are indications of spin-glass-like behavior that result from the non-negligible dipole–dipole interactions among ferromagnetic NPs [82,83,84].

## 4. Conclusions

The correlation between the level of Ga^3+^ substitution in CoNiGa-SFMCs and their crystal structures, optical characteristics, and magnetic properties was described. The peculiarities of the phase content and structural parameters of SFMs were verified using XRD and HR-TEM. The microsphere shape is clearly apparent in the SEM and TEM images. Surface area characterization using nitrogen adsorption isotherms revealed an improvement in the surface area and formation of multimodal pores. The high surface area of CoNiGa-SFMCs (x = 0.6) was 105 m^2^·g^−1^, with an average pore size distribution of approximately 26.2 nm and a pore size volume of 0.68 cm^3^∙g^−1^. The light absorption characteristics of pristine Co_0.5_Ni_0.5_Fe_2_O_4_ SFMCs changed significantly between the (500–900) nm exposed region of incident light for the Ga^3+^ ion-substituted samples. The evaluated direct *E*_g_ magnitudes were about semiconductors for all samples and increased primarily owing to the ion substitution process. Magnetization measurements, versus either the magnetic field or temperature, revealed that all samples displayed ferrimagnetic ordering over the entire 10–300 K temperature range. Ms and $n_{B}$ rose gradually as x raised to 0.6, and then progressively decreased for a much higher x content. However, the H_c_ value showed a decline with a growth in the value of x. Observed variations in the magnetic parameters were linked to variations in crystallite/particle size, cation distribution, and anisotropy. The ZFC and FC magnetization results indicated a presence of spin-glass-like behavior at low temperatures.

## Figures and Tables

**Table 1:** CoNiGa-SFMCs’ cell parameters obtained by Rietveld analysis.

x	*a* (Å)	*V* (Å^3^)	*D_XRD_* (nm) ± 0.04	*χ^2^* (chi^2^)	*R_Bragg_*
**0.0**	8.335 (2)	579.09 (4)	12.36	1.20	1.39
**0.2**	8.344 (8)	581.10 (5)	11.34	1.96	0.70
**0.4**	8.351 (4)	582.48 (3)	11.69	2.15	0.19
**0.6**	8.297 (3)	571.23 (4)	9.99	1.40	0.83
**0.8**	8.326 (5)	577.27 (8)	10.05	2.32	0.27
**1.0**	8.327 (4)	577.46 (2)	9.78	1.58	0.91

**Table 2:** Textural and structural properties of CoNiGa-SFMCs.

x	BET Surface Area (m^2^/g)	Pore Volume (cm^3^/g)	Average Pore Size (nm)
**0.0**	57	0.22	15.8
**0.2**	36	0.13	14.2
**0.4**	51	0.24	18.8
**0.6**	105	0.68	26.2
**0.8**	78	0.32	16.2
**1.0**	79	0.34	17.3

**Table 3:** Main magnetic characteristics of CoNiGa-SFMCs at 10 and 300 K.

x	M_r_ (emu/g)		SR = M_r_/M_s_		K (Oe·emu/g)	
	300 K	10 K	300 K	10 K	300 K	10 K
**0.0**	2.53	32.8	0.085	0.683	1.66 × 10^4^	4.55 × 10^5^
**0.2**	9.38	31.1	0.256	0.555	1.50 × 10^4^	3.77 × 10^5^
**0.4**	4.97	46.4	0.116	0.697	1.04 × 10^4^	4.25 × 10^5^
**0.6**	10.13	59.2	0.193	0.700	1.10 × 10^4^	3.62 × 10^5^
**0.8**	2.19	27.2	0.081	0.539	2.69 × 10^3^	9.30 × 10^4^
**0.1**	1.42	20.2	0.075	0.505	2.12 × 10^3^	1.21 × 10^5^

## Data Availability

Not applicable.
