# Correction: Almessiere et al. Impact of Ga^3+^ Ions on the Structure, Magnetic, and Optical Features of Co-Ni Nanostructured Spinel Ferrite Microspheres. *Nanomaterials* 2022, *12*, 2872

**DOI:** 10.3390/nano16150919

**Published:** 2026-07-27

**Authors:** Munirah A. Almessiere, Yassine Slimani, Sadaqat Ali, Abdulhadi Baykal, Rabindran Jermy Balasamy, Sadik Guner, İsmail A. Auwal, Alex V. Trukhanov, Sergei V. Trukhanov, Ayyar Manikandan

**Affiliations:** 1 Department of Biophysics, Institute for Research and Medical Consultations (IRMC), Imam Abdulrahman Bin Faisal University, P.O. Box 1982, Dammam 31441, Saudi Arabia; 2 Department of Physics, College of Science, Imam Abdulrahman Bin Faisal University, P.O. Box 1982, Dammam 31441, Saudi Arabia; 3 Mechanical and Energy Engineering Department, College of Engineering, Imam Abdulrahman Bin Faisal University, P.O. Box 1982, Dammam 31441, Saudi Arabia; sadali@iau.edu.sa; 4 Department of Nanomedicine Research, Institute for Research and Medical Consultations (IRMC), Imam Abdulrahman Bin Faisal University, P.O. Box 1982, Dammam 31441, Saudi Arabia; abaykal@iau.edu.sa (A.B.); rjermy@iau.edu.sa (R.J.B.); 5 Institute of Inorganic Chemistry, RWTH Aachen University, 52074 Aachen, Germany; s.guner@ac.rwth-aachen.de; 6 Department of Chemistry, Sule Lamido University, Kafin Hausa 731, Nigeria; sahiburrahmah@gmail.com; 7 Smart Sensors Laboratory, Department of Electronic Materials Technology, National University of Science and Technology (MISiS), 119049 Moscow, Russia; truhanov86@mail.ru; 8 Laboratory of Magnetic Films Physics, SSPA Scientific and Practical Materials Research Centre of NAS of Belarus, 19, P. Brovki Str., 220072 Minsk, Belarus; 9 Department of Chemistry, Bharath Institute of Higher Education and Research, Bharath University, Chennai 600073, Tamil Nadu, India; mkavath15@gmail.com

In the original publication [1], there was a mistake in Figure 1 as published. Due to technical image compression and layout rendering constraints during the final multi-step file transfer and publishing processes, the fine details of the experimental patterns and Rietveld refinement profiles in Figure 1 suffered from resolution degradation. This compression introduced artificial visual artifacts, such as apparent local profile steps and distorted minima, which did not accurately reflect the high quality of the true underlying graphical data and fitting parameters.

To ensure complete scientific clarity and technical transparency, Figure 1 has been replaced with the original, uncompressed high-resolution image. This updated figure accurately showcases the fine structural details of both the experimental data (red dots) and the smooth calculated refinement models (black solid lines). The difference profiles between the experimental data and the Rietveld refinement (blue solid lines) for each composition were also included at the bottom of each pattern, alongside the green Bragg position bars, confirming the exceptionally high quality and correctness of the Rietveld fitting parameters.

The authors state that the structural, morphological, magnetic, and optical findings remain fully verified, and the core scientific conclusions of the manuscript are entirely unaffected. The goodness of fit and associated statistical parameters reported in Table 1 remain completely accurate and validated by the original raw data. The corrected Figure 1 is provided below. This correction was approved by the Academic Editor. The original publication has also been updated.

## Figures and Tables

**Figure 1:**
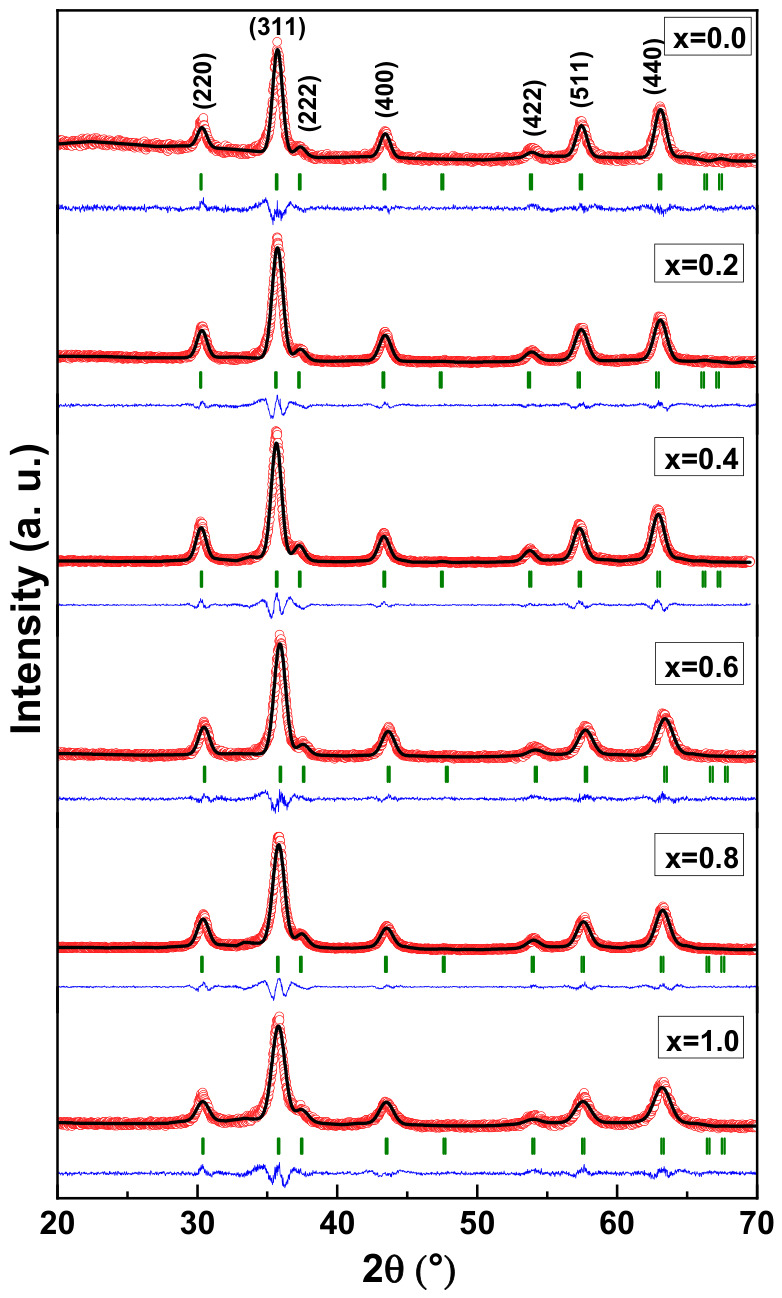
CoNiGa-SFMC XRD powder diffraction patterns. Red dots, black solid lines, blue solid lines, and green bars represent the experimental data, calculated refinement models, difference profiles, and Bragg positions, respectively.
